# Can AI-based body composition assessment outperform body surface area in predicting dose-limiting toxicities for colonic cancer patients on chemotherapy?

**DOI:** 10.1007/s00432-023-05227-7

**Published:** 2023-08-04

**Authors:** Ke Cao, Josephine Yeung, Yasser Arafat, CheukShan Choi, Matthew Y. K. Wei, Steven Chan, Margaret Lee, Paul N. Baird, Justin M. C. Yeung

**Affiliations:** 1 Department of Surgery, Western Precinct, University of Melbourne, Melbourne, Australia; 2 Department of Colorectal Surgery, Western Health, Melbourne, Australia; 3 Department of Oncology, Western Health, Melbourne, Australia; 4 Department of Surgery, University of Melbourne, Melbourne, Australia

**Keywords:** Chemotherapy toxicity, Body composition, Artificial intelligence, Clinical oncology, Computed tomography

## Abstract

**Purpose:**

Gold standard chemotherapy dosage is based on body surface area (BSA); however many patients experience dose-limiting toxicities (DLT). We aimed to evaluate the effectiveness of BSA, two-dimensional (2D) and three-dimensional (3D) body composition (BC) measurements derived from Lumbar 3 vertebra (L3) computed tomography (CT) slices, in predicting DLT in colon cancer patients.

**Methods:**

203 patients (60.87 ± 12.42 years; 97 males, 47.8%) receiving adjuvant chemotherapy (Oxaliplatin and/or 5-Fluorouracil) were retrospectively evaluated. An artificial intelligence segmentation model was used to extract 2D and 3D body composition measurements from each patients' single mid-L3 CT slice as well as multiple-L3 CT scans to produce a 3D BC report. DLT was defined as any incidence of dose reduction or discontinuation due to chemotherapy toxicities. A receiver operating characteristic (ROC) analysis was performed on BSA and individual body composition measurements to demonstrate their predictive performance.

**Results:**

A total of 120 (59.1%) patients experienced DLT. Age and BSA did not vary significantly between DLT and non-DLT group. Females were significantly more likely to experience DLT (p = 4.9 × 10^–3^). In all patients, the predictive effectiveness of 2D body composition measurements (females: AUC = 0.50–0.54; males: AUC = 0.50–0.61) was equivalent to that of BSA (females: AUC = 0.49; males: AUC = 0.58). The L3 3D skeletal muscle volume was the most predictive indicator of DLT (AUC of 0.66 in females and 0.64 in males).

**Conclusion:**

Compared to BSA and 2D body composition measurements, 3D L3 body composition measurements had greater potential to predict DLT in CRC patients receiving chemotherapy and this was sex dependent.

**Supplementary Information:**

The online version contains supplementary material available at 10.1007/s00432-023-05227-7.

## Introduction

Body-surface area (BSA) calculations have been the mainstay for calculating chemotherapy dosing (Drami 2021) for patients with colorectal cancer (CRC). However, many CRC patients receiving chemotherapy experience significant toxic side effects and dose-limiting toxicities (DLT) (Health 2017). This may necessitate dose reduction or discontinuation of their treatment, ultimately resulting in decreased cancer treatment efficacy and suboptimal clinical outcomes (Starobova and Vetter 2017; Arafat 2022).

There is growing evidence in the literature to support the observation that body composition may offer an improved measure in the prediction of chemotherapy induced toxicities (Guo 2021; da Silva Dias 2021; Arafat et al. 2022; Drami et al. 2021). Currently, the majority of studies measuring body composition have examined the surface area of muscle and adipose tissues derived from only a single cross-sectional CT slice at the lumbar 3 vertebral (L3) level (typically from the mid-point of L3, referred to as mid-L3 from hereon) (Cespedes Feliciano 2017; Kurk 2019; Barret 2014; Blauwhoff-Buskermolen 2016). Beyond this, there is a paucity of data in the literature to compare the utility of a mid-L3 CT slice (2D body composition) with multiple CT slices (3D body composition) in the determination of chemotherapy induced toxicities in CRC.

Thus, the objective of this study was to examine and compare the predictive ability of BSA, 2D and 3D body composition measures for the prediction of DLT in CRC patients.

## Methods

This was a single-site, cross-sectional analysis of colonic cancer patients treated with adjuvant Oxaliplatin and Fluorouracil-based chemotherapy regimens at a tertiary referral centre, Western Health in Melbourne, Australia. This study was approved by the Ethics Department at the Western Health Office for Research (Project QA2020.24_63907). The protocol followed the tenets of the Declaration of Helsinki and all privacy requirements were met.

### Patient population

Colonic cancer patients treated at Western Health between 2012 and 2021 were identified from the Australian Comprehensive Cancer Outcomes and Research Database (ACCORD), a prospectively maintained registry of patients diagnosed with CRC in Victoria.

Patients were included in the study if they were diagnosed with colonic cancer and treated at Western Health with chemotherapy following surgery. Patients with non-metastatic disease were included in the study. Patients included in the study were required to have axial CT scans available prior to chemotherapy and within 6 months prior to their primary surgical resection date or 3 months following surgery.

Patients were excluded if there were suboptimal features in their CT scans that would preclude accurate body composition measurement. For example, due to poor CT scan image quality (e.g. no contrast), or significant extension of subcutaneous adipose tissue (SAT) or skeletal muscle (SM) beyond the CT image. An experienced body composition data operator (author JoY) assessed the overall quality of CT scans of patients based on the mid-L3 CT slice. Patients were also excluded if they had incomplete chemotherapy or clinical information. The flow chart for the study cohort is shown in Supplementary Fig. 1.

### Data collection

The ACCORD database was used to acquire patient demographics including age at the time of diagnosis, gender, and BSA value.

Information regarding DLT was collected from institutional electronic medical records. Our primary endpoint, DLT, was defined as any incidence of dose reduction or treatment discontinuation during any cycle of chemotherapy due to chemotherapy-induced toxicities (Health et al. 2017).

The medical image viewer Synapse 5 (FUJIFILM) was used to obtain all available axial CT scans at the L3 level for each patient (2203 scans in total). Each patient had between 4–46 CT scans (depending on slice thickness that ranged from 1–8 mm) at L3. For each patient, a trained human grader (author JoY) manually selected one CT slice as the most representative of the L3 in line with the Alberta Protocol (https://tomovision.com/SarcopeniaHelp/index.html). This slice referred to as the mid-L3 slice, has generally been considered as the gold standard for analysing body segmentation measures in the majority of CRC studies (Arayne 2023; Brown et al. 2022a, b; Kotti 2022).

Each CT scan obtained was represented as a 512*512-pixel-resolution Digital Imaging and Communications in Medicine (DICOM) image (dose value ranging from 100–140 kVp). Each DICOM file contained the pixel value, intercept, and slope of the CT scan. Using the formula: pixel value * slope + intercept, each pixel/unit on the CT scan was converted to the Hounsfield Unit (HU) scale, which represents a quantitative measure of radiodensity for evaluating CT images (Khan et al. 2014).

### Body composition

A pre-trained AI segmentation model (submitted for publication) was used to automatically segment and measure skeletal muscle (SM), visceral adipose tissue (VAT), and subcutaneous adipose tissue (SAT) in the L3 slice of each patient.

The measurement of body composition obtained from a single mid-L3 CT slice was referred to as 2D body composition, whereas the measurement of body composition that was obtained from multiple CT slices derived from the entire L3 vertebrae was referred to as a 3D body composition.

We included the following 2D body composition measurements in this study at the mid-L3 vertebra: SM surface area (cm^2^), SM radiodensity (HU), VAT surface area (cm^2^), VAT radiodensity (HU), SAT surface area (cm^2^), SAT radiodensity (HU), Skeletal muscle index (SMI, SM surface area (cm^2^)/height(m^2^)), Visceral fat index (VFI, VAT surface area(cm^2^)/height(m^2^)), and the Subcutaneous fat index (SFI, SAT surface area (cm^2^)/height(m^2^)).

We included the following 3D body composition measurements in this study of the entire L3 vertebra: SM volume (cm^3^), Average SM radiodensity (HU), VAT volume (cm^3^), Average VAT radiodensity (HU), SAT volume (cm^3^), Average SAT radiodensity (HU).

The surface area (cm^2^) of a particular body composition was determined using the total surface area within the mid-L3 slice by multiplying the size of a particular body composition by the pixel spacing of the CT scan. The pixel spacing was derived using the information included within each CT DICOM file.

The radiodensity (HU) of a particular body composition tissue was obtained using data from the mid-L3 slice by averaging the pixel values representing that body composition.

The volume (cm^3^) of a certain body composition was determined using all L3 slices of a patient using the formula: (sum (surface area of a specific body composition for each slice * slice thickness)).

The average radiodensity (HU) of a certain body composition tissue was determined using all L3 slices of a patient using the formula: ((sum (radiodensity of the body composition under assessment for each slice)) / the total number of L3 slices of a patient).

### Statistical analysis

Receiver operating characteristic (ROC) analyses were performed for each individual body composition measurement as well as each patient’s BSA to calculate the area under the curve (AUC) (Hanley and McNeil 1982; Scheipers 2005; Prati et al. 2008; Altman and Bland 1994). Based on the AUC value, ROC analysis was used to demonstrate how effectively a parameter discriminated between DLT and no DLT groups (Verbakel 2020). AUC varied from 0 to 1, with 1 indicating perfect accuracy of classification. The ROC analysis was undertaken using the ‘roc’ function from the 'pROC' package in RStudio (version 2022.2.2.485) for Windows, and the optimal cut-off value for each parameter was determined. A cut-off point dichotomized patients based on a specific measurement, to provide the prediction (DLT or no DLT) (Unal 2017). Based on concurrent evaluation of sensitivity and specificity, a cut-off point was deemed optimal when it correctly classified the majority of individuals. The resulting AUC values were then compared to assess the prediction performance of each individual parameter.

The prediction based on the parameter with the highest AUC values was determined, and the accuracy, sensitivity and specificity were calculated for this prediction (Zhu et al. 2010). Accuracy was determined by dividing the number of patients correctly classified by the total number of patients. Specificity represents the prediction's ability to accurately identify DLT from the given DLT group, whereas specificity represents the prediction's ability to accurately identify non-DLT from the given non-DLT group under investigation.

Statistical analyses were performed using RStudio (version 2022.2.2.485) for Windows. Statistical tests were deemed significant if their p-value was less than 0.05. The Mann–Whitney test was used to compare continuous parameters (i.e. all body composition measurements and age) between groups. The Chi-squared test was used to evaluate categorical variables, i.e. gender.

## Results

A total of 203 colonic cancer patients were available for inclusion in the study, including 97 males (47.8%) and 106 females (52.2%). The overall mean age was 60.87 ± 12.42 years with males (62.78 ± 12.12 years) being significantly older than females (59.11 ± 12.48 years). 120 patients (59.1%) experienced DLT, with females significantly more likely to experience DLT (73/106, 69%) compared with male (47/97, 48%) patients (p = 4.9 × 10^–3^).

Age, BSA and body composition characteristics of females and males with and without DLT are shown in Table 1. There were no significant differences in BSA, age, or measures of 2D body composition between the DLT and no DLT groups.

**Table 1:** Age, BSA and body compositions characteristics of females and males, with and without DLT

	Females (n = 106 patients)			Males (n = 97 patients)		
	Median (Interquartile range)			Median (Interquartile range)		
	DLT (n = 73 patients)	No DLT (n = 33 patients)	P-value	DLT (n = 47 patients)	No DLT (n = 50 patients)	P-value
Age	62.00 (51.00–68.00)	60.00 (50.00–65.00)	0.29	65.00 (56.00–70.50)	65.00 (54.00–73.75)	0.96
BSA (m^2^)	1.76 (1.64–1.91)	1.78 (1.60–1.88)	0.87	1.90 (1.72–2.00)	1.91 (1.78–2.10)	0.15
SM						
2D (mid-L3)						
Surface area^a^	102.33 (94.27–117.36)	107.67 (97.35–119.95)	0.64	148.90 (136.05–160.59)	146.72 (134.31–164.62)	1
Radiodensity^b^	37.41 (29.18–42.71)	34.57 (31.25–43.84)	0.88	41.21 (34.33–44.84)	37.09 (32.15–40.59)	0.07
3D (complete L3)						
Volume^c^	313.2 (277.0–354.5)	358.9 (322.4–401.4)	**0.011**	443.3 (381.2–501.1)	479.0 (441.7–583.0)	**0.015**
Radiodensity	36.94 (28.72–41.75)	34.92 (31.50–42.24)	0.78	40.97 (34.13–44.18)	36.89 (31.82–41.06)	0.07
VAT						
2D (mid-L3)						
Surface area	109.36 (70.18–174.35)	102.86 (68.60–174.97)	0.81	178.24 (85.05–289.07)	197.40 (125.37–265.73)	0.64
Radiodensity	− 90.58 (− 95.88–− 84.15)	− 90.26 (− 94.84–− 86.36)	0.96	− 91.15 (− 95.16–− 83.52)	− 91.58 (− 97.96–− 85.56)	0.51
3D (complete L3)						
Volume	359.83 (206.01–521.12)	318.1 (241.6–525.8)	0.89	532.91 (250.03–808.42)	653.68 (410.80–878.50)	0.31
Radiodensity	− 90.83 (− 95.81–− 84.36)	− 90.87 (− 95.16–− 85.01)	1	− 91.51 (− 95.85–− 83.38)	− 91.05 (− 97.70–− 85.72)	0.57
SAT						
2D (mid-L3)						
Surface area	264.26 (191.97–347.31)	261.36 (155.02–343.74)	0.65	145.36 (93.43–176.77)	159.5 (115.5–248.6)	0.22
Radiodensity	− 104.00 (− 107.95–− 99.93)	− 103.82 (− 107.77–− 101.16)	0.97	− 97.52 (− 104.14–− 91.94)	− 100.60 (− 104.20–− 92.60)	0.44
3D (complete L3)						
Volume	785.9 (541.1–1021.2)	745.5 (505.9–1229.0)	0.66	446.90 (299.38–549.72)	534.98 (384.65–846.29)	0.053
Radiodensity	− 103.90 (− 107.57–− 100.66)	− 104.6 (− 106.7–− 100.8)	0.94	− 97.59 (− 104.21–− 91.80)	− 100.44 (− 104.93–− 92.32)	0.38

^a^Unit for surface area was cm^2^

^b^Unit for radiodensity was HU

^c^Unit for volume was cm^3^

### 2D body composition analysis

The predictive performance of 2D body composition measurements in identifying patients who had developed DLT was evaluated. ROC curves for classifying subjects in the DLT or no DLT groups were determined for each individual 2D body composition measurement and BSA. The AUC and corresponding optimal cut-point given by each individual 2D body composition measurement and by BSA are shown in Table 2.

**Table 2:** Area under ROC curves for predicting DLT utilising BSA and each 2D body composition measurement in males and females respectively

Parameters	Females (n = 106 patients)		Male (n = 97 patients)	
	AUC (95% CI)	Optimal cut-point (threshold)	AUC (95% CI)	Optimal cut-point (threshold)
SM				
BSA	0.49 (0.37–0.61)	1.82	0.58 (0.47–0.70)	2.09
Mid-L3 surface area	^**a**^**0.53** **(0.41–0.65)**	110.08	0.50 (0.38–0.62)	141.46
SMI	^**a**^**0.54** **(0.43–0.66)**	40.34	0.50 (0.39–0.62)	43.24
Mid-L3 radiodensity	^**a**^**0.51** **(0.39–0.63)**	34.61	^**a**^**0.61** **(0.49–0.72)**	41.03
VAT				
Mid-L3 surface area	^**a**^**0.52** **(0.39–0.64)**	131.42	0.53 (0.41–0.65)	118.48
VFI	^**a**^**0.52** **(0.39–0.64)**	42.66	0.52 (0.40–0.64)	41.95
Mid-L3 radiodensity	^**a**^**0.50** **(0.38–0.61)**	− 95.70	0.54 (0.42–0.65)	− 97.20
SAT				
Mid-L3 surface area	^**a**^**0.53** **(0.40–0.65)**	148.90	0.57 (0.46–0.69)	185.56
SFI	^**a**^**0.52** **(0.40–0.65)**	73.31	0.57 (0.46–0.69)	63.76
Mid-L3 radiodensity	^**a**^**0.50** **(0.38–0.62)**	− 110.64	0.55 (0.43–0.66)	− 97.73

^a^Body composition measurements with an AUC greater than BSA are highlighted in bold

In female patients, all 2D body composition measures demonstrated a slightly higher predictive ability (AUC 0.50–0.54) for DLT compared with BSA (AUC = 0.49). In male patients, only SM radiodensity exhibited a higher predictive value (AUC = 0.61) than BSA (AUC = 0.58) in predicting DLT.

### 3D body composition analysis

The 3D L3 SM volume in DLT patients was significantly lower than that in non-DLT patients for both female (p = 0.011) and male groups (p = 0.015) (Table 1). No other significant differences in any of the other 3D body composition measures were noted between the DLT and no DLT groups.

The predictive performance of 3D body composition measurements in identifying patients who had developed DLT was evaluated. Table 3 displays the AUC and corresponding optimal cut-point for each 3D body composition. Skeletal muscle (SM) volume was the most predictive body composition measure of DLT with an AUC of 0.66 and 0.64 compared to the AUC of BSA of 0.49 and 0.58 in females and males respectively. ROC curves for prediction of DLT and no DLT based on SM volume in females and males were shown in supplementary Fig. 2 and 3. In male patients, the predictive performance of average SM radiodensity (AUC = 0.61) and SAT volume (AUC = 0.61) were also both superior compared to using BSA alone (AUC = 0.58).

**Table 3:** Area under the curve (ROC analysis) for predicting DLT comparing BSA and different 3D body composition measurements in males and females respectively

Parameters	Female (n = 106 patients)		Male (n = 97 patients)	
	AUC (95% CI)	Optimal cut-point (threshold)	AUC (95% CI)	Optimal cut-point (threshold)
BSA	0.49 (0.37–0.61)	1.82	0.58 (0.47–0.70)	2.09
SM				
L3 volume	^**a**^**0.66** (0.54–0.77)	352.17	^**a**^**0.64** (0.53–0.75)	437.07
L3 average radiodensity	0.52 (0.40–0.64)	35.74	^**a**^**0.61** (0.49–0.72)	41.26
VAT				
L3 volume	0.49 (0.37–0.62)	337.34	0.56 (0.44–0.68)	353.75
L3 average radiodensity	0.50 (0.38–0.62)	− 86.68	0.47 (0.35–0.58)	− 66.17
SAT				
L3 volume	0.47 (0.35–0.60)	387.08	^**a**^**0.61** (0.50–0.73)	542.07
L3 average radiodensity	0.50 (0.39–0.62)	− 104.62	0.55 (0.44–0.67)	− 105.10

^a^Body composition measurements with a ROC greater than 0.6 highlighted in bold

**Fig. 1:**
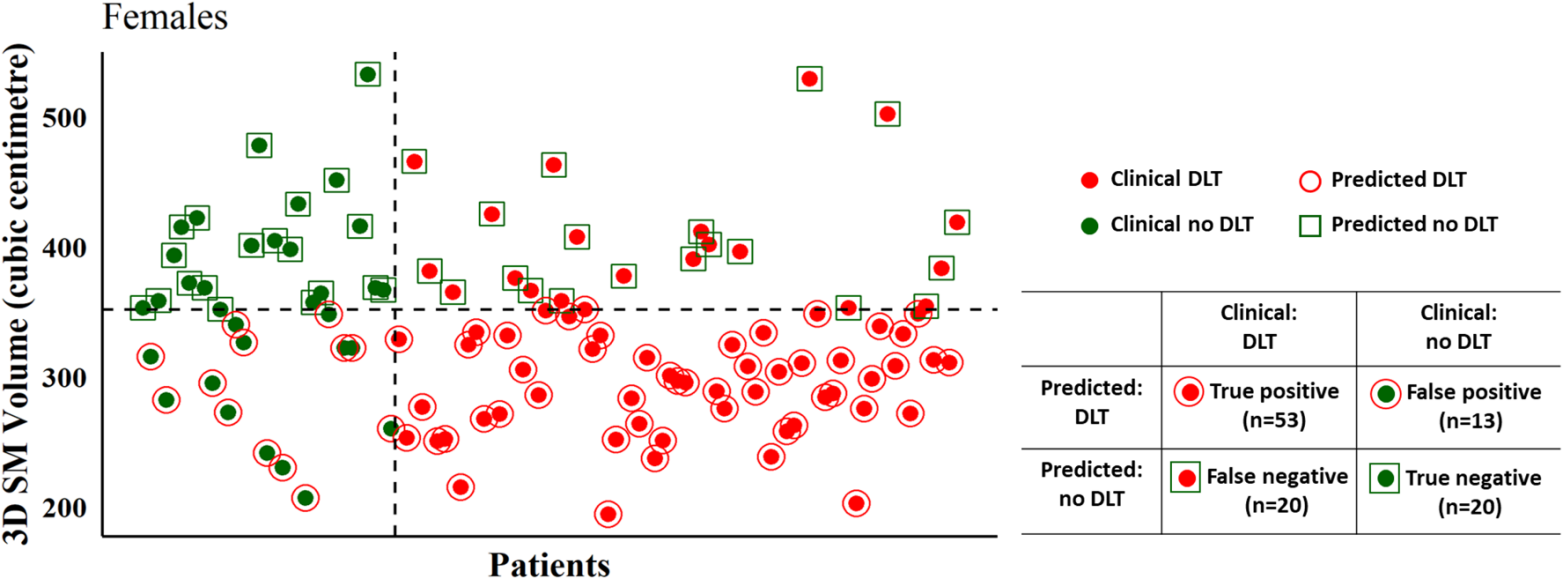
Performance metrics comparing current clinical classification with 3D SM volume-based prediction of DLT and non-DLT in female patients

**Fig. 2:**
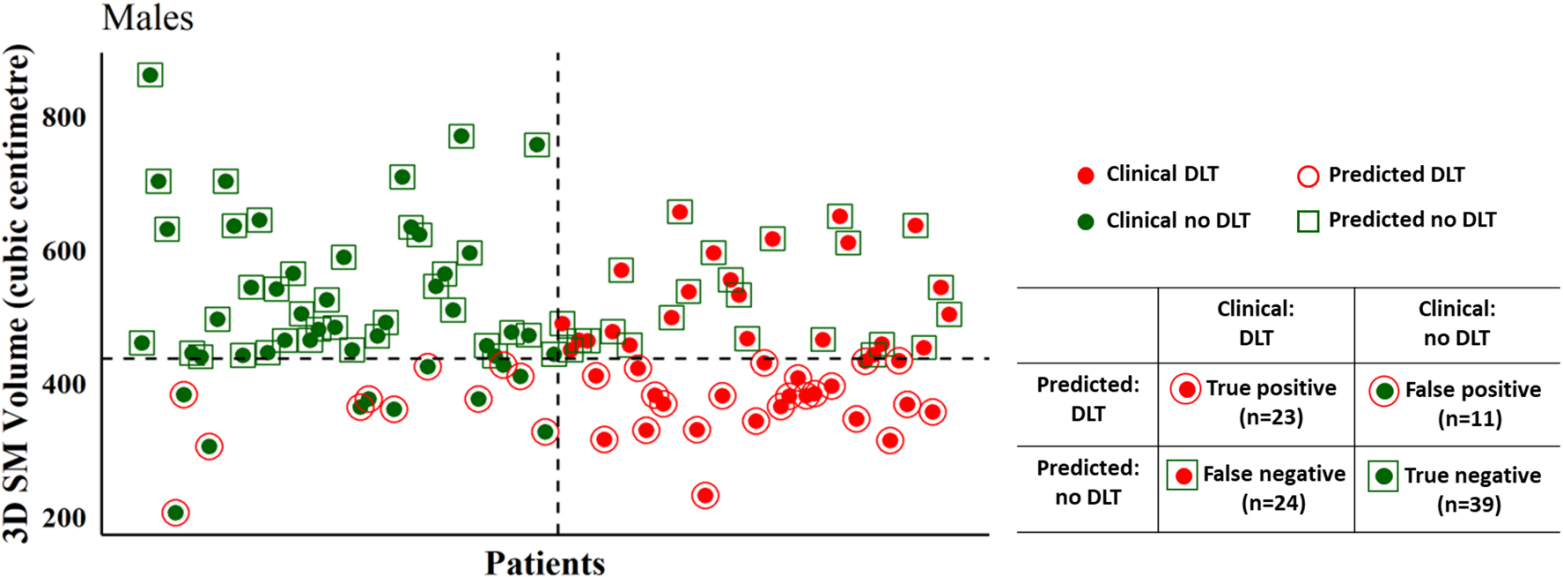
Performance metrics comparing current clinical classification with 3D SM volume-based prediction of DLT and non-DLT in male patients

Following ROC analysis, the cut-off values for the SM volume were determined to be 352.17 cm^3^ for females and 437.07 cm^3^ for males respectively. These cut-off values identified 73/106 (53/73 DLT patients, and 20/33 no DLT) for an overall prediction accuracy of 0.69 (sensitivity = 0.73, specificity = 0.61) in female patients (Fig. 1). In male patients, 3D SM volume-based prediction correctly classified 62/97 (23/47 DLT patients, and 39/50 no DLT) to achieve an overall accuracy of 0.64 (sensitivity = 0.49, specificity = 0.78) in predicting DLT (Fig. 2).

## Discussion

Adjuvant chemotherapy is indicated in the treatment of advanced colonic cancer. The current dosing of these agents is mainly based on patient BSA. However, patients with similar BSA and of different sex have been shown to exhibit different side effect profiles due to variations in body composition (Ali 2016).

We have shown that BSA was poor at predicting DLT in colonic cancer patients, where almost three-fifths of our patients (59.1%) developed severe toxicities. We have also shown that body composition measurements derived from SM, VAT, and SAT may be more accurate predictors of DLT than BSA. Oxaliplatin and 5FU are both hydrophilic in nature and the volume of distribution is correlated with lean muscle mass and both mainly metabolised by the liver. It has also been known that sarcopenia is related to reduced completion of oxaliplatin-based regimens in patients with CRC and is strongly associated with severe chemotherapy toxicity in patients with metastatic CRC (Shiraishi 2023; Barret 2014). In addition, patients who are obese have been shown to have an increased risk of chemotherapy toxicities, suggesting that the distribution pattern and overall quantity of adipose tissue (SAT and VAT) have a significant impact on colonic cancer chemotherapy storage and metabolism (Cespedes Feliciano 2017; Brown 2022a, b; Shiraishi et al. 2023; Jung 2015). These findings have highlighted the crucial role body composition may play in determining whether or not a patient will experience DLT. However, using validated cut-offs for sarcopenia based on Prado et al. (2008) reveals limited accuracy in predicting DLT (accuracy of 0.45 for males and 0.53 for females, supplementary table 1), indicating that a new approach to predicting is required.

Previous studies in the literature primarily investigated the association between 2D body composition and DLT (Lee 2015; Looijaard 2020; Cespedes Feliciano et al. 2017; Brown et al. 2022a, b; Shiraishi et al. 2023; Jung et al. 2015; Drami et al. 2021). This was primarily due to the lack of AI algorithms that could perform segmentation on large datasets. In these older studies, researchers reported conflicting findings with some studies reporting a statistically significant association between muscle and/or adipose tissue and DLT (Cespedes Feliciano et al. 2017; Brown et al. 2022a, b; Shiraishi et al. 2023; Jung et al. 2015), whereas others found the opposite (Lee et al. 2015; Looijaard et al. 2020). An explanation could be that results may differ when analysing different chemotherapy regimens and employing different definitions of DLT and body composition thresholds within the same study. In addition, other studies attempted to determine the optimal lean body mass (LBM) threshold for predicting DLT in colon cancer patients (Prado 2007; Ali 2016). Using logistic regression, an earlier study (Prado et al. 2007) analysed 62 patients receiving 5FU-based chemotherapy and found that 20 mg 5-FU/kg LBM appeared to be a threshold for developing DLT. Another study (Ali et al. 2016) analysed 138 patients receiving oxaliplatin-based chemotherapy and reported a cut point of only 3.09 mg oxaliplatin/kg LBM using a ROC analysis. Due to the absence of performance metrics in the studies, we are unable to compare the prediction results from these studies directly.

To the best of our knowledge, this is the first study comparing the predictive power of 2D and complete 3D lumbar 3 CT-derived body composition parameters with BSA regarding chemotherapy toxicity in colon cancer. Our findings have demonstrated that all 2D body composition parameters in females had a higher AUC than BSA; whereas only SM radiodensity had a higher AUC than BSA in males. The results from our cohorts indicated that the HU of VAT has a slightly higher predictive ability than its surface area in males. Furthermore, when examining 3D entire lumbar 3 vertebra body composition measurements, total 3D SM volume was a superior body composition predictor compared to both 2D body composition measurements and BSA in identifying patients who developed DLT. It is important to note that the 3D SM volume provided a considerably different DLT prediction for males and females. The sensitivity of using 3D SM volume in predicting DLT in females was 73%, indicating a 73% likelihood of accurately predicting DLT patients. However, the sensitivity of using 3D SM volume was considerably lower (0.49) in male patients. This was also mirrored in the ROC analysis where AUC was 0.61 (accuracy = 0.62) in males but only 0.47 (accuracy = 0.54) in females. Despite being the greatest predictor of DLT for both females and males, 3D SM volume has distinct performance characteristics. It may be administered to predict female patients who are likely to develop toxicity (high sensitivity) and to predicting male patients who are unlikely to develop toxicity (high specificity).

Several limitations of our study should be acknowledged. In particular, we only used a retrospective patient dataset from a single tertiary referral centre and our numbers were relatively low. However, there was minimum bias around treatment decision making and follow up data retention was high due to the prospective colorectal registry which our institution has utilised over the last decade. We agree that definitions of toxicity which allowed us to measure DLT had an inherent bias due to the retrospective nature of our study. We therefore decided to define DLT based on any situation where the patient had a reduction or cessation of chemotherapy; however, we did not consider other types of toxicities or stratify patients based on specific complications; these are anticipated to be included in future studies. In addition, although recent CT scans were utilised in the analysis of body composition, we acknowledge that body parameters can vary even over a short period of time in the post- operative period.

## Conclusion

Body surface area has a limited ability to predict chemotherapy-induced DLT in colonic cancer patients. The use of 2D body composition measurements at L3 improved accuracy slightly. The most predictive findings were obtained using 3D body composition measures at L3 to identify patients at risk of DLT, however, its utility was sex dependent. The incorporation of 3D body composition measurements into the development of a predictive tool may help in the future with personalised chemotherapy dosing. More investigations are required to determine the optimal method for reducing DLT and dosing chemotherapeutics based on body composition, and future research should include drug pharmacokinetics.

### Supplementary Information

Below is the link to the electronic supplementary material.

Supplementary file1 (DOCX 108 KB)

Supplementary file2 (DOCX 13 KB)

## Data Availability

The datasets generated during and/or analysed during the current study are available from the corresponding author on reasonable request.
