# Supplementary material for: Can AI-based body composition assessment outperform body surface area in predicting dose-limiting toxicities for colonic cancer patients on chemotherapy?
Source: J Cancer Res Clin Oncol. 2023 Aug 4;149(15):13915–23. doi: 10.1007/s00432-023-05227-7 (PMC10590342; doi:10.1007/s00432-023-05227-7)
Supplement: Supplementary file 1 — Supplementary file1 (DOCX 108 KB) [file 432_2023_5227_MOESM1_ESM.docx]

**Can AI-Based Body Composition Assessment Outperform Body Surface Area in Predicting Dose-Limiting Toxicities for Colonic Cancer Patients on Chemotherapy?**

**Journal of Cancer Research and Clinical Oncology**

**Authors:**

Ke Cao ^1^, Josephine Yeung ^1^, Yasser Arafat ^1,2^, CheukShan Choi ^1^, Matthew YK Wei ^1,2^, Steven Chan ^1^, Margaret Lee ^3^, Paul N Baird ^4#^, Justin MC Yeung ^1,2#, a^

# These authors contributed equally as senior authors

**Affiliations:**

1 Department of Surgery, Western Precinct, University of Melbourne, Australia, 2 Department of Colorectal Surgery, Western Health, Melbourne, Australia, 3 Department of Oncology, Western Health, Melbourne, Australia, 4 Department of Surgery, University of Melbourne, Australia

**^a^Correspondence:**

Professor Justin Yeung

Consultant Colorectal Surgeon

Head of the Department of Surgery, Western Precinct

University of Melbourne

Level 3, WCHRE Building, Sunshine Hospital

T: +61 3 8395 8116 E: justin.yeung@unimelb.edu.au

**
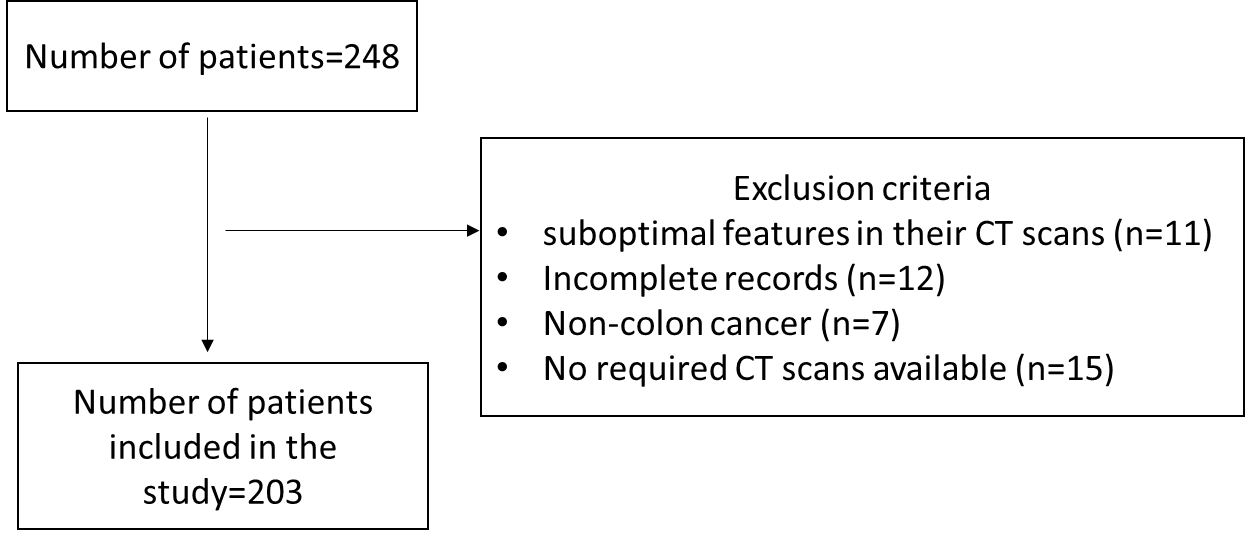
**

**Supplementary Figure 1 Flowchart of study cohort.**


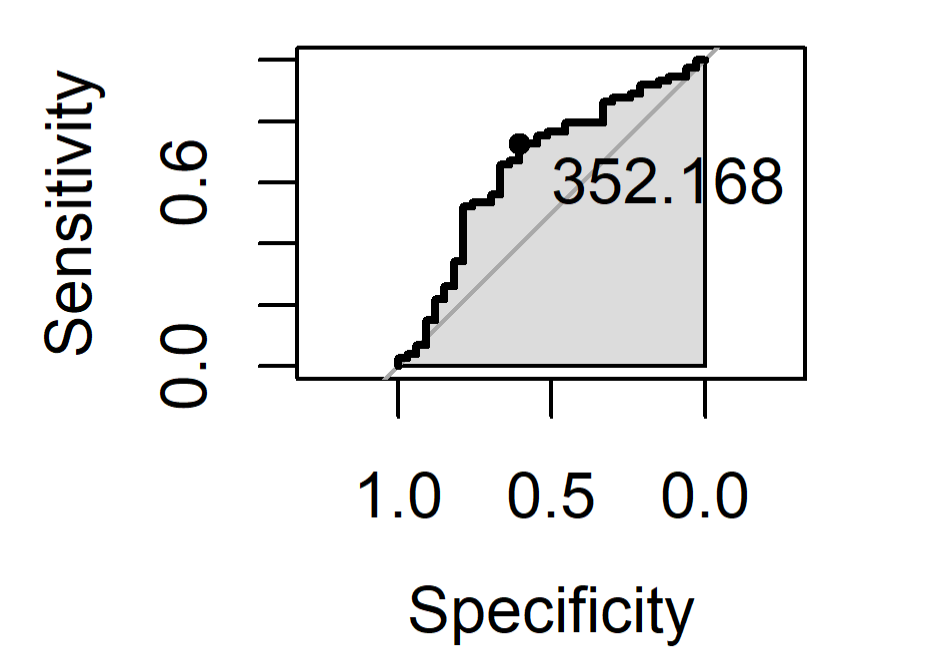


**Supplementary Figure 2 ROC curve for prediction of DLT and no DLT based on SM volume in females.**

**
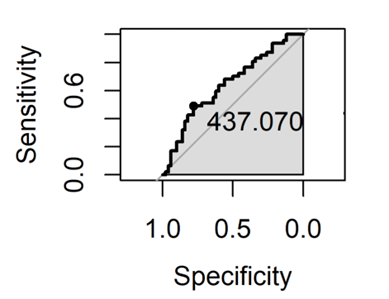
**

**Supplementary Figure 3 ROC curve for prediction of DLT and no DLT based on SM volume in males.**
