# Supplementary material for: Can AI-based body composition assessment outperform body surface area in predicting dose-limiting toxicities for colonic cancer patients on chemotherapy?
Source: J Cancer Res Clin Oncol. 2023 Aug 4;149(15):13915–23. doi: 10.1007/s00432-023-05227-7 (PMC10590342; doi:10.1007/s00432-023-05227-7)
Supplement: Supplementary file 2 — Supplementary file2 (DOCX 13 KB) [file 432_2023_5227_MOESM2_ESM.docx]

**Supplementary Table 1 Predicting DLT utilising validated cutoffs for sarcopenia in males and females respectively. Patients with sarcopenia were identified using the SMI threshold established by Prado et al. An SMI value of <38.5 cm²/m² in women and <52.4 cm²/m² in men was categorised as being sarcopenic.**

|  | Female | | Male | |
| --- | --- | --- | --- | --- |
|  | No DLT | DLT | No DLT | DLT |
| No sarcopenia | 23 | 48 | 23 | 19 |
| Sarcopenia | 10 | 25 | 27 | 28 |
